# Structural Evidence for Pnictogen-Centered Lewis Acidity in Cationic Platinum-Stibine Complexes Featuring Pendent Amino or Ammonium Groups †

**DOI:** 10.3390/molecules26071985

**Published:** 2021-04-01

**Authors:** Roberta R. Rodrigues, François P. Gabbaï

**Affiliations:** Department of Chemistry, Texas A&M University, College Station, TX 77843-3255, USA; bertacarbene@tamu.edu

**Keywords:** pnictogen bonding, platinum, catalysis

## Abstract

As part of our continuing interest in the chemistry of cationic antimony Lewis acids as ligands for late transition metals, we have now investigated the synthesis of platinum complexes featuring a triarylstibine ligand substituted by an *o*-[(dimethylamino)methyl]phenyl group referred to as Ar^N^. More specifically, we describe the synthesis of the amino stibine ligand Ph_2_SbAr^N^ (**L**) and its platinum dichloride complex [**L**PtCl]Cl which exists as a chloride salt and which shows weak coordination of the amino group to the antimony center. We also report the conversion of [**L**PtCl]Cl into a tricationic complex [**L**HPt(SMe_2_)]^3+^ which has been isolated as a tris-triflate salt after reaction of [**L**PtCl]Cl with SMe_2_, HOTf and AgOTf. Finally, we show that [**L**HPt(SMe_2_)][OTf]_3_ acts as a catalyst for the cyclization of 2-allyl-2-(2-propynyl)malonate.

## 1. Introduction

**Figure 1:**
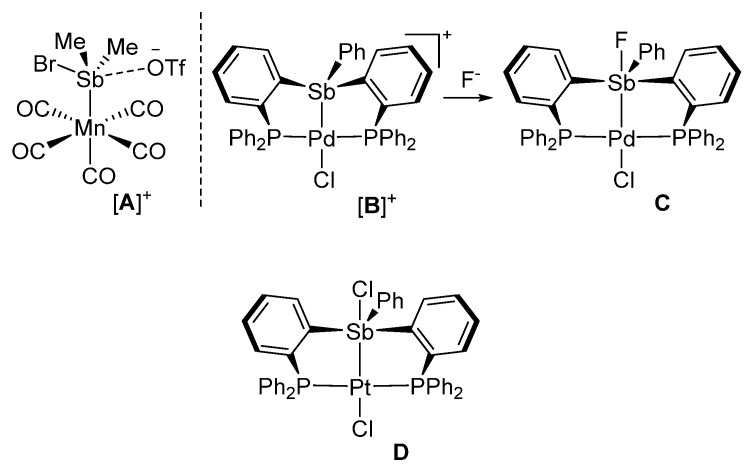
Structure of representative stibine complexes in which the antimony atom displays Lewis acidic properties.

The chemistry of stibines is attracted a renewed interest because of applications in material science and catalysis [1,2,3,4]. Although a parallel is often drawn with the ubiquitous phosphine ligands, stibines display several atypical traits and can, for example, undergo redox reactions without dissociation of the coordinated metal [5,6,7,8,9,10]. Moreover, while phosphines mostly act as two-electron donor spectator ligands, stibines have been shown to display Lewis acidic properties, even when coordinated to a transition metal [11,12,13,14,15,16,17,18,19,20,21,22]. These Lewis acidic properties manifest in the formation of long contacts between the antimony center of cationic complexes and counteranions as in complex [**A**]**^+^** which was isolated by Reid (Figure 1) [11]. Short contacts, akin to polar covalent bonds, can also be formed in the case of small halide anions, including the fluoride anion [23]. Such behavior comes to the fore in the case of complexes featuring bis(phosphino)stibine ligands such as [**B**]^+^ which is readily converted into **C** in the presence of a fluoride anion (Figure 1) [23].

A previous article described the platinum complex **D**, which features a chloride anion coordinated to the antimony center trans from the platinum atom (Figure 1) [13]. The resulting Sb-Cl bond formed by this complex is ~14% longer than the sum of the covalent radii indicating partial ionicity. This lengthening suggested that the simple addition of a donor functionality would suffice in promoting the dissociation of this anion, providing access to a cationic complex. Therefore, based on this simple hypothesis, we have decided to replace the antimony-bound phenyl group of **D** by an *o*-[(dimethylamino)methyl]phenyl group. This substituent is known to form intramolecular N-Sb bonds when incorporated in antimony derivatives [24,25] as demonstrated over two decades ago by Cowley, Jones, and Norman [26] as well as by Akiba [27]. In this paper, we report the results of our efforts along with the conversion of the target complex in a triflate derivative that behaves as a carbophilic catalyst.

## 2. Results and Discussion

**Scheme 1:**
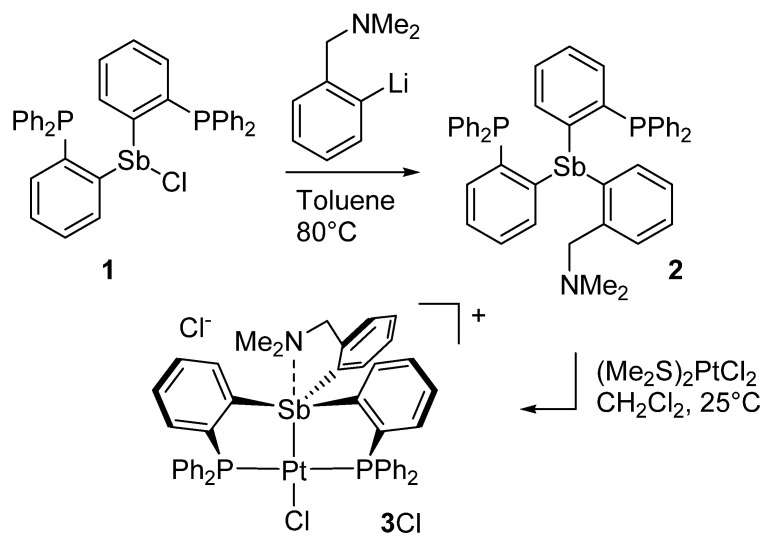
Synthesis of ligand 2 and its conversion into the corresponding platinum(II) complex [**3**][Cl].

As a first step, we decided to introduce the *o*-[(dimethylamino)methyl]phenyl group by reaction of the corresponding lithium salt [27] with the bis(phosphino)chlorostibine **1** [28] as shown in Scheme 1. The reaction was carried out in toluene upon heating to 80 °C. Following standard workup and column chromatography, the new amino-functionalized ligand **2** was obtained as an air-stable solid in 71% yield The presence of the *o*-[(dimethylamino)methyl]phenyl group could be easily confirmed by ^1^H NMR spectroscopy, which displays resonances at 1.69 ppm and 3.37 ppm (in C_6_D_6_) corresponding to the benzylic methylene and dimethylamino groups, respectively. Integration of these two resonances produced the expected intensity ratio when referenced to the resonances arising from the phenylene protons in the aromatic region of the spectrum. The presence of the phosphino groups was easily confirmed by the detection of a single resonance at −8.19 ppm in the ^31^P NMR spectrum.

With this ligand at our disposal, we decided to investigate its reaction with (Me_2_S)_2_PtCl_2_. This reaction, which was carried out in CH_2_Cl_2_ at room temperature, proceeded smoothly to afford complex [**3**][Cl] as an air-stable solid in 89% yield. The 31P NMR spectrum of complex [**3**][Cl] features a resonance at 49.52 ppm that displays the expected 195Pt satellites. The 1JPt−P coupling constant of 2524 Hz is comparable to that measured for complex D (1JPt−P = 2706 Hz), suggesting the formation of a divalent square planar platinum complex. Other notable spectroscopic features include a downfield of the phenylene proton positioned ortho from the antimony atom from 7.50 ppm in 2 to 8.37 ppm in [**3**][Cl]. Finally, the benzylic methylene and dimethylamino group give rise to sharp resonances at 1.71 ppm and 3.10 ppm, respectively. Because the chemical shift of these resonances is close to those in the free ligand 2, we resorted to X-ray analysis to elucidate the antimony atom’s coordination environment in [**3**][Cl] and the eventual presence of a dative N→Sb dative interaction.

**Figure 2:**
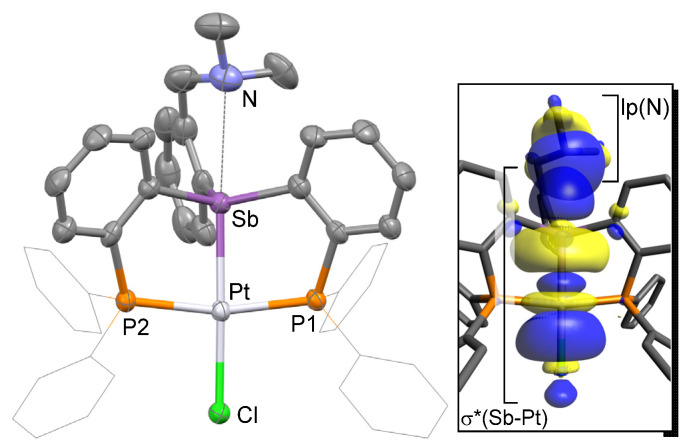
Solid-state structure of complex [3][Cl]. Thermal ellipsoids are drawn at the 50% probability level. The hydrogen atoms, interstitial solvent molecules, and counteranions are omitted for clarity while the Ph groups are draw as thin lines. Relevant metrical parameters can be found in the text.

Single crystals of complex [**3**][Cl] could be easily obtained upon diffusion of pentane into a CH_2_Cl_2_ solution of [**3**][Cl] at ambient temperature in air. Complex [**3**][Cl] crystallized in the *P*2_1_/*c* space group with one molecule in the asymmetric unit (Table 1, Figure 2). Inspection of the solid-state structure indicated the formation of a cationic complex formed by complete ionization of the Sb–Cl bond, resulting in the presence of a chloride counter anion sitting in the lattice and forming a hydrogen bond with an adventitious water molecule. The cationic complex [**3**]^+^ possesses a platinum atom in a square planar geometry with a chloride ligand coordinated trans from the antimony atom as in the case of **D** [13]. The resulting Sb–Pt bonds of 2.4928(5) Å is notably shorter than that in **D** (2.5380(8) Å). This shortening can be correlated to the absence of an antimony-bound chloride ligand in the case of [**3**][Cl]. Indeed, at first glance, the antimony moiety of [**3**]^+^ corresponds to a rather classical platinum-coordinated stibine unit. In support of this formulation, we note that the Sb–Pt bond in [**3**][Cl] is comparable to that found in classical coordination complexes such as *cis*-[PtCl_2_(SbPh_3_)_2_] (av. 2.50 Å) [29]. The Pt–Cl distance of 2.3621(15) Å is slightly shorter than that in **D** (2.3851(16) Å), indicating that the platinum center of [**3**][Cl] is slightly more electrophilic than in **D**. Finally, we note that the nitrogen of the amino group sits at 2.962(7) Å from the antimony center.

**Figure 3:**
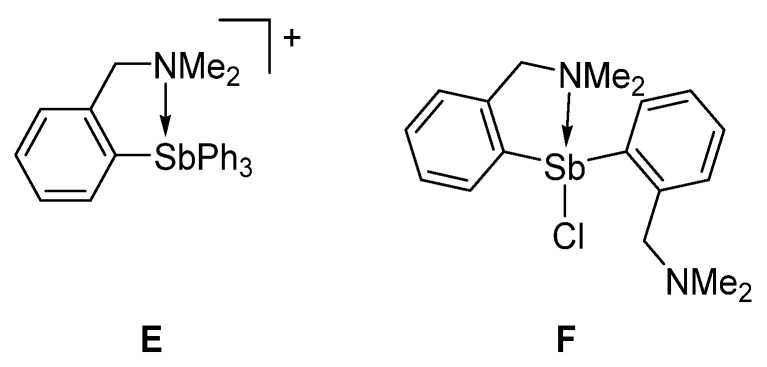
Selected examples of derivatives with N→Sb dative interactions.

This distance is longer than that measured in **E** [30] or **F** [26], which possess N→Sb dative interactions of 2.693(11) Å and 2.463(2) Å, respectively (Figure 3). Based on this comparison, we conclude that an N→Sb dative interaction is also present in [**3**][Cl], even if weaker than in **E** and **F**. Efforts to reproduce the experimental geometry with DFT methods were not successful and led to computed structures where the Sb–N distance was overestimated by ~0.3 Å. We assign our difficulty in reproducing this distance to its inherent weakness. An optimization carried out with the MPW1PW91 functional, a mixed basis set, and application of a solvation model afforded a N–Sb distance of 3.26 Å. Despite this elongation, an NBO analysis of the bonding indicates the persistence of an N→Sb dative interaction that accounts for *E*_del_ = 8.0 kcal/mol to the stability of the molecule (Figure 2, Figure S11).

This distance is longer than that measured in **E** [30] or **F** [26], which possess N→Sb dative interactions of 2.693(11) Å and 2.463(2) Å, respectively (Figure 3). Based on this comparison, we conclude that an N→Sb dative interaction is also present in [**3**][Cl], even if weaker than in **E** and **F**. Efforts to reproduce the experimental geometry with DFT methods were not successful and led to computed structures where the Sb–N distance was overestimated by ~0.3 Å. We assign our difficulty in reproducing this distance to its inherent weakness. An optimization carried out with the MPW1PW91 functional, a mixed basis set, and application of a solvation model afforded a N–Sb distance of 3.26 Å. Despite this elongation, an NBO analysis of the bonding indicates the persistence of an N→Sb dative interaction that accounts for *E*_del_ = 8.0 kcal/mol to the stability of the molecule (Figure 2, Figure S11).

Given these results, we became eager to verify if this interaction was nonetheless sufficient to promote dissociation of the chloride anion from the antimony atom in solution. To this end, we decided to carry out conductance measurements in CH_2_Cl_2_ solution. In keeping with literature precedents, we decided to carry out these measurements with solution containing 1 mM concentration of the species [31]. We first benchmarked our technique using Ph_3_SbCl_2_ and [Ph_4_Sb][OTf] [32], which afforded molar conductivities (Λ_Μ_) of 0.26 and 48.4 S∙cm^2^ mol^−1^, respectively. These results indicated that Ph_3_SbCl_2_ does not dissociate under these conditions while [Ph_4_Sb][OTf] behaves as a 1:1 electrolyte. Next, we compared complex **D** and [**3**][Cl] and observed a contrasting behavior. Indeed while **D** shows a low conductivity 18.52 S cm^2^ mol^−1^, [**3**][Cl] displays a molar conductance of 52.4 S∙cm^2^ mol^−1^, close to that of [Ph_4_Sb][OTf] indicating that [**3**][Cl] is also 1:1 electrolyte. To ascertain this conclusion, we also measured the conductance of [Ph_4_Sb][OTf] and [**3**][Cl] at 0.5 and 0.1 mmol. Via extrapolation of the resulting data points to infinite concentrations, we obtained Λ° values of 138 S cm^2^ mol^−1^ for [Ph_4_Sb][OTf] and 120 S cm^2^ mol^−1^ for [**3**][Cl]. These values match the range determined for similar 1:1 electrolytes in organic solvents [33]. These experiments indicate that, despite the weakness of the N–Sb interaction, the presence of a dimethylamino-methylene group was sufficient to drive the heterolytic dissociation of the Sb–Cl bond both in solution as well as in the solid-state.

**Figure 4:**
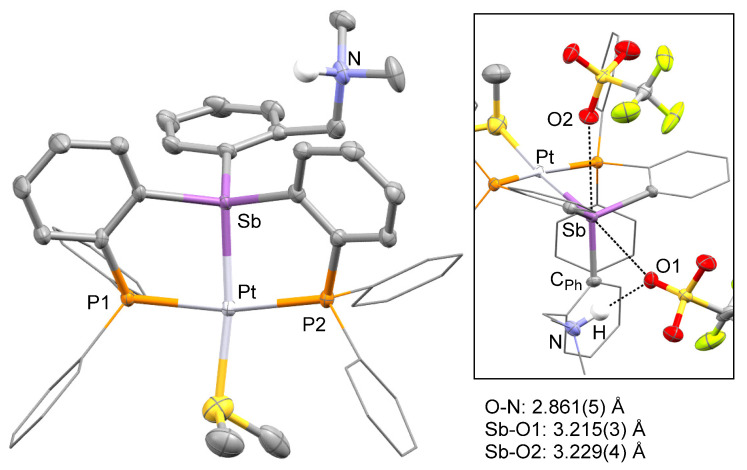
Solid-state structure of complex [**4**][OTf]_3_. Thermal ellipsoids are drawn at the 50% probability level. The hydrogen atoms, interstitial solvent molecules, and counteranions are omitted for clarity while the Ph groups are draw as thin lines. The inset shows weak interactions involving the triflate anions along with selected distances. Other relevant metrical parameters can be found in the text.

In prior studies, we have observed that antimony platinum triflate complexes are endowed with attractive carbophilic reactivity [34,35]. Encouraged by these recent findings, we attempted to convert [**3**][Cl] into the corresponding bis(triflate) derivative. This reaction proved inconsistent and failed to deliver the targeted bis(triflate). In one of these reactions, which was carried out by treating a crude batch of **2** with AgOTf, a small crop of crystals of a new complex was isolated. X-ray analysis of these crystals revealed the formation of a tricationic complex isolated as a triflate salt (Table 1, Figure 4). The structure of this new salt, referred to as [**4**][OTf]_3_, also showed protonation of the nitrogen atom as well as the presence of a dimethylsulfide ligand bound to the platinum center. Because of its protonation, the nitrogen atom is no longer involved in a donor–acceptor interaction with the antimony atom. The latter adopts a distorted tetrahedral geometry and is coordinated to the square planar platinum atom, trans from the site occupied by the Me_2_S ligand. The resulting Sb–Pt distance of 2.4929(3) Å is almost identical to that in [**3**][Cl]. The dimethylsulfide ligand is bound to the platinum atom via a Pt-S distance of 2.3864(2) Å which can be compared to the value of 2.3611(8) Å measured in the cationic complex [κ^2^-2-*N*Me_2_-3-*P^i^*Pr_2_-indene)PtMe(SMe_2_)]^+^ [36]. Finally, the ammonium group forms a hydrogen bonding interaction with one of the triflate counter anions (Figure 4). This triflate counter anion is also involved in a long Sb–O contact of 3.215(3) Å, along the direction of the Sb–Pt bond. Another triflate anion approaches the antimony center along the direction defined by the Sb–C_Ph_ bond, leading to another Sb–O contact of 3.229(4) Å. These contacts are reminiscent of those observed in the structure of complexes such as **A**.

**Scheme 2:**
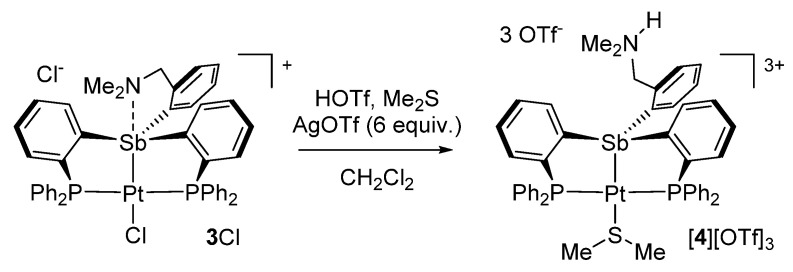
Synthesis of [**4**][OTf]_3_.

The presence of dimethylsulfide in [**4**][OTf]_3_ suggested it was present as an impurity in the crude batch of **2** used in the experiment. Aiming for a reproducible and higher-yielding synthesis of [**4**][OTf]_3_, [**3**][Cl] was treated with AgOTf (6 equiv.) in the presence of triflic acid and dimethylsulfide (Scheme 2). This procedure afforded [**4**][OTf]_3_ in a 45% yield. The ^31^P NMR spectrum of [**4**][OTf]_3_ features a single resonance at 50.65 ppm coupled to the ^195^Pt nuclei by ^1^*J*_Pt−P_ = 3020 Hz. This value, although larger than that for [**3**][Cl] remains consistent with the existence of a divalent platinum center. In the ^1^H NMR spectrum, the phenylene proton positioned ortho from the antimony atom appears at 8.75 ppm, a value that can be compared to that of 8.37 ppm measured for [**3**][Cl]. The benzylic methylene group is observed at 2.78 ppm, while the nitrogen-bound methyl group gives rise to a doublet (^3^*J*_H-H_ = 5.0 Hz) at 2.07 ppm. The ammonium proton gives rise to a broad signal at 6.94 ppm. Freshly prepared crystals of [**4**][OTf]_3_ show a ^1^H NMR resonance at 1.76 ppm assigned to the dimethylsulfide ligand. The coordination of this ligand to the platinum center was confirmed by the detection of ^195^Pt satellites spaces by a ^3^*J*_Pt-H_ coupling constant of 43.9 Hz. This value is comparable to that of 36.5 Hz reported for [κ^2^-2-*N*Me_2_-3-*P^i^*Pr_2_-indene)PtMe(SMe_2_)]^+^ [36].

**Scheme 3:**
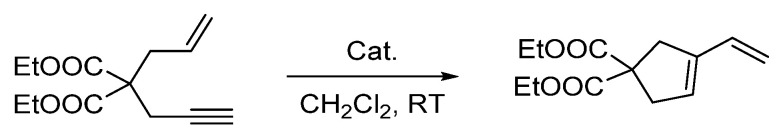
Enyne cyclization reaction used to benchmark the catalytic activity of the complexes.

To conclude this study, we tested the catalytic activity of [**4**][OTf]_3_ in the cycloisomerization of 2-allyl-2-(2-propynyl)malonate, a reaction type well known to be promoted by electrophilic platinum catalysts [19,34,37,38]. We found that the reaction proceeded in CD_2_Cl_2_ when carried out in a sealed NMR tube at 50 °C (Scheme 3). With a catalyst loading of 5 mol%, monitoring of the reaction by ^1^H NMR spectroscopy showed the formation of the five-membered isomer with a conversion greater than 90% after 3 h (see Figure S10). When the same reaction was attempted with [**3**][Cl], no conversion was observed even after 24 h, at the same temperature. This difference underscored the advantages gained by introduction of triflate counterions. Finally, we will note the parallel that exists between [**4**][OTf]_3_ and [(*o*-(Ph_2_P)C_6_H_4_)_2_Sb(OTf)_2_]PtCl (**G**), which also promoted such enyne cyclization reactions, albeit at room temperature [34]. The higher temperature needed in the case of [**4**][OTf]_3_ is tentatively assigned to the presence of a Me_2_S ligand which tames the reactivity of the platinum center.

## 3. Materials and Methods

### 3.1. General Experimental Considerations

Compound **1** and PtCl_2_(SMe_2_)_2_ was prepared according to the reported procedure [28,39]. Toluene and Et_2_O were dried by reflux under N over Na/K and distilled before use. MeCN and CH_2_Cl_2_ were dried over CaH_2_ and distilled before use. Pentane was dried over activated molecular sieves. All other solvents were used as received. Commercially available chemicals were purchased and used as provided (Commercial sources: Sigma-Aldrich, St. Louis, MO, USA for SbCl_3_; Matrix Scientific, Columbia, SC, USA for AgOTf). Ambient temperature NMR spectra were recorded on a Bruker Avance 400 or 500 FT NMR (499.42 MHz for ^1^H, 125.62 MHz for ^13^C, 469.89 MHz for ^19^F, 202.16 MHz for ^31^P). ^1^H and ^13^C NMR chemical shifts are given in ppm and are referenced against SiMe_4_ using residual solvent signals used as secondary standards. Elemental analyses (EA) were performed at Atlantic Microlab (Norcross, GA, USA).

### 3.2. Synthesis of Compound **2**

Compound **1** (344 mg, 0.51 mmol) and (2-((dimethylamino)methyl)phenyl)lithium were dissolved in toluene (2 mL). The resulting mixture was heated to 80 °C for 12 h. The solution was filtered, and the solvent evaporated. The crude product was purified by column chromatography using 10–20% ethyl acetate/hexanes to afford **2** as a white solid (280 mg, 71% yield). ^1^H NMR (400 MHz; C_6_D_6_): δ 7.60–7.34 (m, 3H), 7.35–7.25(m, 9H), 7.02–6.95 (m, 19H), 6.90 (t, 1H, ^3^*J*_H–H_ = 8.0 Hz), 3.37 (s, 2H), 1.69 (s, 6.00 H). ^13^C{^1^H} NMR (125.62 MHz; C_6_D_6_): δ 153.2 (dd, *J*_C-P_ = 55.7 Hz), 144.22 (s), 144.17 (d, *J*_C-P_ = 4.8 Hz), 142.80 (t, *J*_C-P_ = 12.9 Hz),138.12 (s), 137.66 (d, *J*_C-P_ = 14.1 Hz), 137.05 (d, *J*_C-P_ = 14.1 Hz), 136.42 (d, *J*_C-P_ = 14.1 Hz), 133.30 (d, *J*_C-P_ = 20.7 Hz), 133.12 (s), 132.67 (d, *J*_C-P_ = 20.7 Hz), 128.23 (s), 64.92 (s), 43.17 (s). ^31^P{^1^H} NMR (161.74 MHz; C_6_D_6_): δ −8.19. This compound was not subjected to combustion analysis. The reported yield was thus a crude yield. The NMR spectra of this derivative are available in Figures S1–S3.

### 3.3. Synthesis of [**3**][Cl]

Compound **2** (527 mg, 0.68 mmol) and PtCl_2_(SMe_2_)_2_ (245 mg, 0.63 mmol) were combined in CH_2_Cl_2_ (2 mL). After stirring at room temperature for 12 h, the solvent was removed under vacuum and the crude residue was dissolved in CH_2_Cl_2_ (1 mL). The resulting solution was added dropwise to 10 mL of Et_2_O which resulted in the precipitation of [**3**][Cl] as a light yellow solid. This compound was isolated by filtration and dried under vacuum. This procedure afforded [**3**][Cl] in a crude yield of 96% yield (630 mg). ^1^H NMR (400 MHz; CDCl_3_): δ 8.37 (d, 2H, *o*-P(Sb)C_6_H_4_, ^3^*J*_H–H_ = 8.0 Hz), 7.90 (t, 2H, ^3^*J*_H–H_ = 8.0 Hz), 7.67–7.52 (m, 6H), 7.50–7.28 (m, 16H), 7.27–7.24 (m, 3H), 7.01 (d, 1H, ^3^*J*_H–H_ = 8.0 Hz), 6.90 (d, 1H, ^3^*J*_H–H_ = 8 Hz), 6.82 (t, 1H, ^3^*J*_H–H_ = 8.0 Hz), 3.10 (s, 2H), 1.71 (s, 6H). ^13^C{^1^H} NMR (125.62 MHz; CDCl_3_): δ 141.06 (s), 140.51 (t, *J*_C-P_ = 19.7 Hz), 138.94 (t, *J*_C-P_ = 30.3 Hz), 136.77 (s), 136.12 (brs), 135.76 (t, *J*_C-P_ = 9.2 Hz), 134.06 (m), 132.72 (brs), 132.28 (s), 132.18 (s), 131.88 (s), 129.46 (t, *J*_C-P_ = 5.6 Hz), 128.94–128.60 (m), 128.34 (s), 128.13 (brs), 127.91 (s), 127.66 (s), 126.52 (s), 63.02 (s), 45.87 (s). ^31^P{^1^H} NMR (161.74 MHz; CDCl_3_): δ 49.52 (s, ^1^*J*_Pt–P_ = 2706 Hz). The compound could be recrystallized by diffusion of pentane into a CH_2_Cl_2_ of [**3**][Cl] at ambient temperature in air. This procedure afforded crystals of [**3**][Cl] with two interstitial molecules of CH_2_Cl_2_ and one molecule of water as established by X-ray diffraction. A portion of the recrystallized sample was subjected to combustion analysis, which afforded the following results. Elemental analysis calcd (%) for C_45_H_40_Cl_2_NP_2_PtSb-H_2_O-0.5(CH_2_Cl_2_): C: 49.46, H: 3.92; found: C 49.50, H: 3.95. These combustion analysis results suggested the presence of interstitial water and CH_2_Cl_2_, with the latter being partly depleted, presumably as a result of solvent loss during shipping. The NMR spectra of this derivative are available in Figures S4–S6.

### 3.4. Synthesis of [**4**][OTf]_3_

Salt [**3**][Cl] (100 mg, 0.10 mmol) was dissolved in CH_2_Cl_2_ (1ml) and treated with AgOTf (148 mg, 0.58 mmol) under an N_2_ atmosphere. The reaction mixture was allowed to stir for 2 h then one drop of trifluoromethanesulfonic acid and one drop of dimethyl sulfite was added to the reaction mixture. The reaction mixture was then allowed to stir for 12 h. The AgCl precipitate was filtered with 0.45 um PTFE syringe filter and the product was isolated by vapor diffusion of Et_2_O (2 mL) into MeCN to yield colorless crystals (70 mg, 48% yield) suitable for X-ray diffraction. ^1^H NMR (400 MHz; CD_3_CN): δ 8.92 (d, 2H, *o*-P(Sb)C_6_H_4_, ^3^*J*_H–H_ = 8.0 Hz), 8.20 (brs, 1H) 7.96 (t, 2H, ^3^*J*_H–H_ = 8.0 Hz), 7.92–7.81 (m, 4H), 7.80–7.56 (m, 21H), 7.45–7.37(m, 2H), 6.70 (t, 1H, ^3^*J*_H–H_ = 4.0 Hz), 6.12 (d, 1H, ^3^*J*_H–H_ = 8.1 Hz), 2.76 (d, 6H, ^3^*J*_H–H_ = 5.0 Hz), 2.20 (d, 6H, ^3^*J*_H–H_ = 5.0 Hz), 1.76 (s, 6H, ^3^*J*_Pt–H_ = 43.9 Hz). ^13^C{^1^H} NMR (125.62 MHz; CDCl_3_): δ 142.02 (t, *J*_C-P_ = 34.0 Hz), 137.89 (t, *J*_C-P_ = 7.6 Hz), 136.46 (brs), 135.79 (t, *J*_C-P_ = 7.7 Hz), 135.46 (s), 135.31–134.98 (m), 134.64 (brs), 134.56 (s), 134.36 (t, *J*_C-P_ = 6.64 Hz), 134.06 (s), 132.90 (s), 132.85 (s), 131.61 (s), 131.56 (s), 131.53 (s), 130.47 (t, *J*_C-P_ = 5.6 Hz), 126.12 (t, *J*_C-P_ = 29.8 Hz), 125.50 (t, *J*_C-P_ = 29.6 Hz), 122.15 (s), 119.60 (s), 117.05 (s), 116.94 (s), 60.13 (s), 42.84 (s), 23.21 (s). ^31^P{^1^H} NMR (161.74 MHz; CDCl_3_): δ 50.65 (s, ^1^*J*_Pt–P_ = 3020 Hz). Elemental analysis calcd (%) for C_48_H_41_F_9_NO_9_P_2_PtS_3_Sb: C: 40.47, H: 3.9; found: C 40.38, H: 3.32. The NMR spectra of this derivative are available in Figures S7–S9.

### 3.5. Conductance Measurements

Conductance measurements were carried out in CH_2_Cl_2_ at concentrations using a Mettler Toledo FiveGo conductivity probe. All compounds were first measured at 1 mM concentrations. In the case of [Ph_4_Sb][OTf] and [**3**][Cl], conductance measurements were carried out at three concentrations affording: at 1 mM, 0.5 mM, and 0.1 mM, affording the following conductance values: 48.4 μS cm^−1^ at 1 mM, 28.8 μS cm^−1^ at 0.5 mM, and 9.22 μS cm^−1^ at 0.1 mM for Ph_4_SbOTf; 52.4 μS cm^−1^ at 1 mM, 29.0 μS cm^−1^ at 0.5 mM, and 8.4 μS cm^−1^ at 0.1 mM for [3][Cl]. The Λ° molar conductivity values were obtained by plotting the conductance data as a function of the square root of the concentration and by extrapolating to infinite dilution.

### 3.6. Crystallographic Measurements

The crystallographic measurements were performed at 110(2) K using a Bruker Venture diffractometer ([**3**][Cl]) (Cu K_α_ radiation, λ = 1.54178 Å) and a Bruker Quest diffractometer for [**4**][OTf]_3_ (Mo K_α_ radiation, λ = 0.71073 Å). In each case, a specimen of suitable size and quality was selected and mounted onto a nylon loop and cooled to 110(2) K in a cold nitrogen stream (OXFORD Crysosystems). The data were collected and reduced using Bruker AXS APEX 3 software [40] and solved by direct methods. Semiempirical absorption corrections were applied using SADABS [41]. Subsequent refinement was carried with the SHELXTL/PC package (version 6.1 & OLEX^2^) [42,43]. Thermal parameters were refined anisotropically for all non-hydrogen atoms to convergence. Hydrogen atoms were added at idealized positions using a riding model. CCDC 2067400 ([**3**][Cl]) and 2067401 ([**4**][OTf]_3_) contains the supplementary crystallographic data for this paper. These data can be obtained free of charge via http://www.ccdc.cam.ac.uk/conts/retrieving.html (or from the CCDC, 12 Union Road, Cambridge CB2 1EZ, UK; Fax: +44-1223-336033; E-mail: deposit@ccdc.cam.ac.uk.

### 3.7. DFT Calculations

The structure of complex [**3**]^+^ was optimized using DFT methods (see Table S1 for resulting atomic coordinates) in Gaussian 09 [44]. In all cases, the crystal structure of derivative was chosen as a starting point and the Polarizable Continuum Model (PCM) (solvent = water) was applied during the optimization. The following level of theory was employed: MPW1PW91 functional and a mixed basis set (cc-pVTZ-PP for Pt and Sb; 6-311G(d,p) for H/C/N/P/Cl). Frequency calculations, performed at the same level of theory with application of the PCM (solvent = acetonitrile), found no imaginary frequencies. NBO analysis was performed with the NBO 6.0 program [45] using the same level of theory as in the optimization.

## 4. Conclusions

The results presented in this study show that the inclusion of a pendent dimethylamino group positioned next to the antimony center of [**3**][Cl] promotes chloride anion dissociation from the antimony center, facilitating cation formation. As indicated by the isolation of [**4**][OTf]_3_, this amino group can also be readily protonated without compromising the PtSb dinuclear core’s integrity. Although the carbophilic reactivity of [**4**][OTf]_3_ suffers from the presence of a Me_2_S ligand bound to the platinum center, the inclusion of a donor amino functionality may present advantages. Indeed, we speculate that such a functionality could relay protons during catalysis and possibly facilitate protodematellation steps, a possibility we are investigating by attempting the incorporation of ligand **2** in complexes with exposed late transition metals. Finally, the electron-deficient characteristics of [**4**][OTf]_3_ manifest in the formation of two long Sb-O_triflate_ contacts speaking to the Lewis acidic or pnictogen-bond donor properties of the antimony atom in this structure.

## Figures and Tables

**Table 1:** Crystal data and structure refinement for [**3**][Cl] and [**4**]OTf_3_^1^.

	[**3**][Cl]-(CH_2_Cl_2_)_2_-(H_2_O)	[**4**]OTf_3_-MeCN
Empirical formula	C_47_H_46_Cl_6_NOP_2_PtSb	C_52_H_50_F_9_N_2_O_9_P_2_PtS_4_Sb
M	1232.33 g/mol	1524.96 g/mol
Temperature	110 K	110 K
Wavelength	1.54178 Å	0.71073 Å
Crystal system	Monoclinic	Monoclinic
Space group	*P*2_1_/*c*	*P*2_1_/*c*
Unit cell dimensions	*a* = 11.0507(5) Å	*a* = 13.2984(15) Å
	*b* = 18.1574(8) Å	*b* = 14.3100(17) Å
	*c* = 24.0847(11) Å *β* = 99.231(2)°	*c* = 30.743(3) Å *β* = 101.601(3)°
Volume	4770.1(4) Å3	5730.9(11) Å3
Z	4	4
Density (calculated)	1.716 g/cm^3^	1.767 g/cm^3^
Absorption coefficient	13.906 mm^−1^	3.196 mm^−1^
Θ range	3.062 to 66.593°.	1.963 to 28.281°.
Reflections collected	56,128	126,808
Independent reflections	8379 [*R*_int_ = 0.0648]	14,206 [*R*_int_ = 0.0639]
Goodness-of-fit on *F*^2^	1.065	1.081
R indices (all data)	*R*_1_ = 0.0494, *wR*_2_ = 0.1206	*R*_1_ = 0.0530, *wR*_2_ = 0.0853

^1^ Additional details are available in the CIF files that have been deposited with the CCCDC.

## Data Availability

The data is available in the Supplementary Materials accompanying this article.

## Supplementary Materials

The following are available online, Figure S1: ^1^H NMR of compound **2** in C_6_D_6_, Figure S2: ^13^C{^1^H} NMR of compound **2** in C_6_D_6_, Figure S3: ^31^P{^1^H} NMR spectrum of compound **2** in C_6_D_6_, Figure S4:^1^H NMR spectrum of compound [**3**][Cl] in CDCl_3_, Figure S5: ^31^P{^1^H} NMR spectrum of compound [**3**][Cl] in CDCl_3_, Figure S6:^13^C{^1^H} NMR spectrum of compound [**3**][Cl] in CDCl_3_, Figure S7:^1^H NMR spectrum of compound [**4**][OTf]_3_ in CD_2_Cl_2_, Figure S8: ^13^C{^1^H} NMR spectrum of compound [**4**][OTf]_3_ in CD_2_Cl_2_, Figure S9: ^31^P{^1^H} NMR spectrum of [**4**][OTf]_3_ in CD_2_Cl_2_, Figure S10: ^1^H NMR spectra of collected during the cyclization of 2-allyl-2-(2-propynyl)malonate catalyzed by [**4**][OTf]_3_ (5 mol%) in CD_2_Cl_2_ at 50 °C, Figure S11: Selected Natural Bond Orbitals involved in the N→Sb interaction present in the [**3**]^+^, Table S1: Cartesian coordinates (in Å) for the optimized structure of [**3**]^+^.

## Sample Availability

The compounds described in this paper were not synthesized in sufficiently large quantities to be available for distribution.
