# Supplementary material for: Structural Evidence for Pnictogen-Centered Lewis Acidity in Cationic Platinum-Stibine Complexes Featuring Pendent Amino or Ammonium Groups
Source: Molecules. 2021 Apr 1;26(7):1985. doi: 10.3390/molecules26071985 (PMC8036533; doi:10.3390/molecules26071985)
Supplement: Supplementary file 1 [file molecules-26-01985-s001.pdf]

Structural evidence for pnictogen-centred Lewis acidity in cationic platinum-stibine complexes featuring pendent amino or ammonium groups

Roberta R. Rodrigues and François P. Gabbaï*

Department of Chemistry, Texas A&M University, College Station, TX 77843-3255.

TABLE OF CONTENT

Figure S1: ^{1}H NMR of compound **2** in C_6D_6....2
Figure S2: ^{13}C{^{1}H} NMR of compound **2** in C_6D_6....2
Figure S3: ^{31}P{^{1}H} NMR spectrum of compound **2** in C_6D_6....3
Figure S4: ^{1}H NMR spectrum of compound [**3**][Cl] in $CDCl_3$....3
Figure S5: ^{31}P{^{1}H} NMR spectrum of compound [**3**][Cl] in $CDCl_3$....4
Figure S6: ^{13}C{^{1}H} NMR spectrum of compound [**3**][Cl] in $CDCl_3$....4
Figure S7: ^{1}H NMR spectrum of compound [**4**][OTf]$_3$ in CD_2Cl_2....5
Figure S8: ^{13}C{^{1}H} NMR spectrum of compound [**4**][OTf]$_3$ in CD_2Cl_2....5
Figure S9: ^{31}P{^{1}H} NMR spectrum of [**4**][OTf]$_3$ in CD_2Cl_2....6
Figure S10: ^{1}H NMR spectra of collected during the cyclization of 2-allyl-2-(2-propynyl)malonate catalyzed by [**4**][OTf]$_3$ (5 mol%) in CD_2Cl_2 at 50°C....6
Figure S11: Selected Natural Bond Orbitals involved in the N→Sb interaction present in the [**3**]$^+$....7
Table S1. Cartesian coordinates (in Å) for the optimized structure of [**3**]$^+$....7

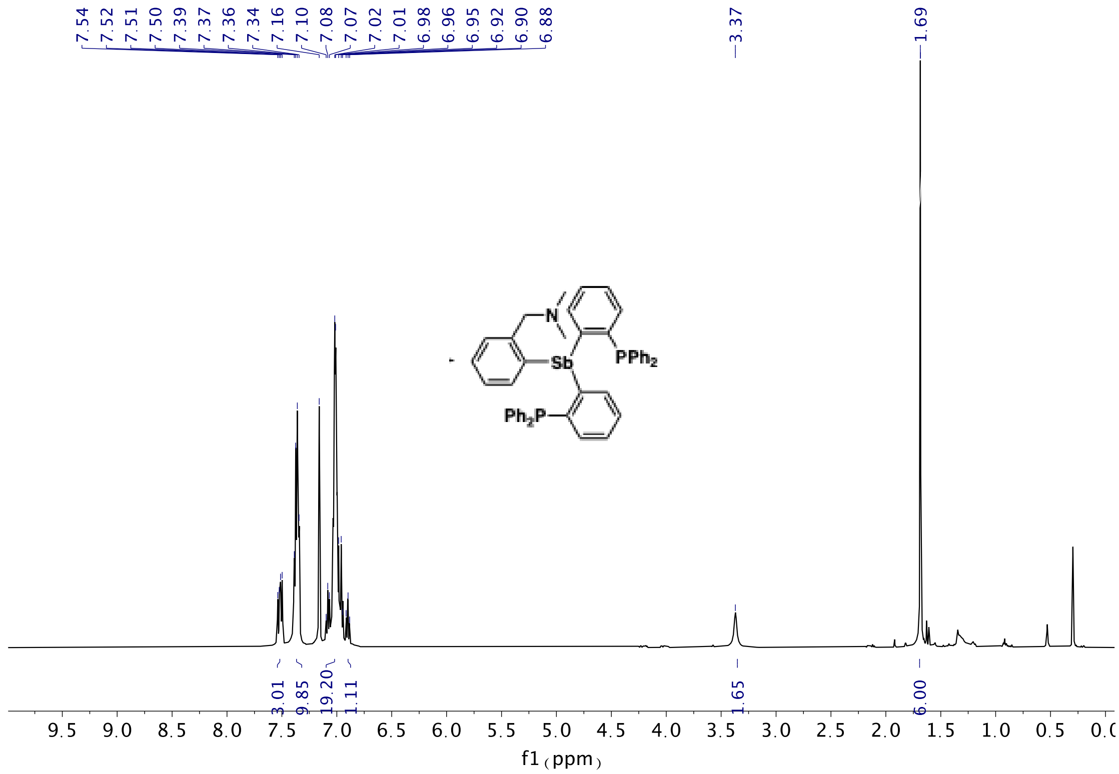

Figure S1: ^{1}H NMR of compound **2** in C_6D_6.

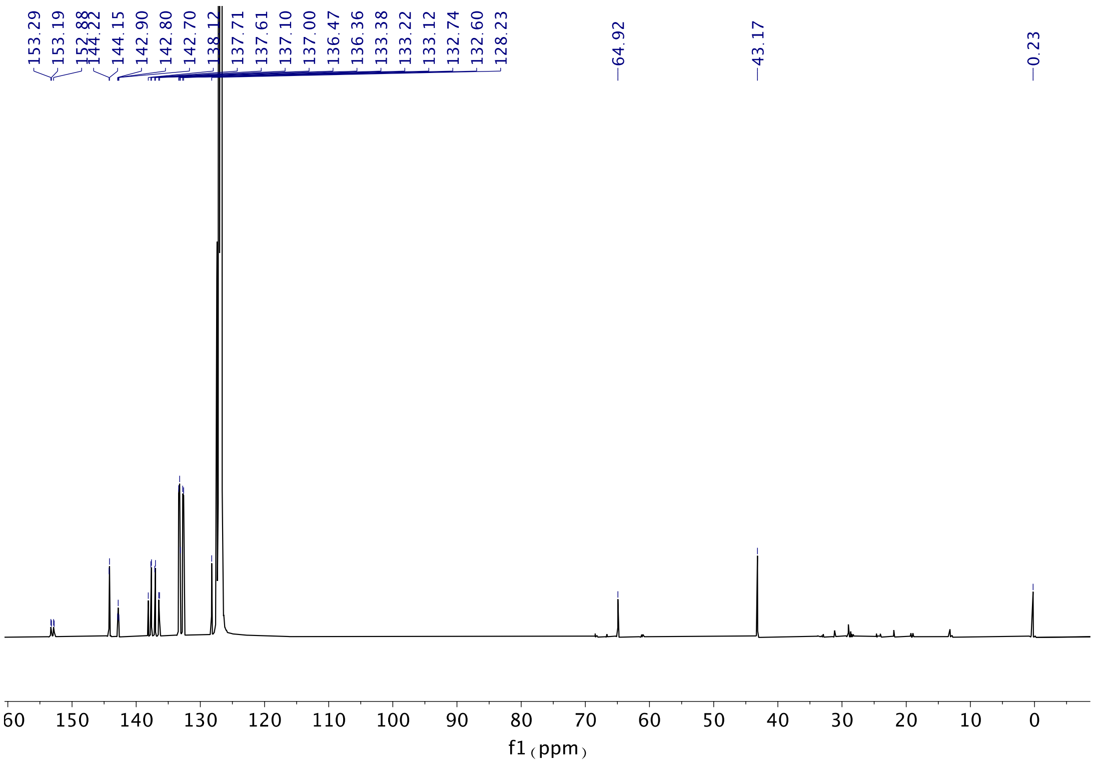

Figure S2: $^{13}C\{^{1}H\}$ NMR of compound **2** in C_6D_6.

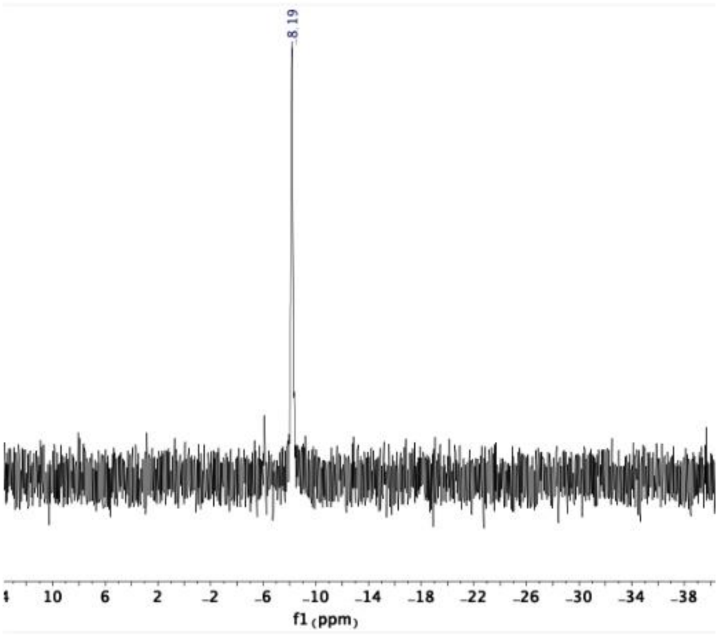

Figure S3: $^{31}P\{^{1}H\}$ NMR spectrum of compound **2** in C_6D_6.

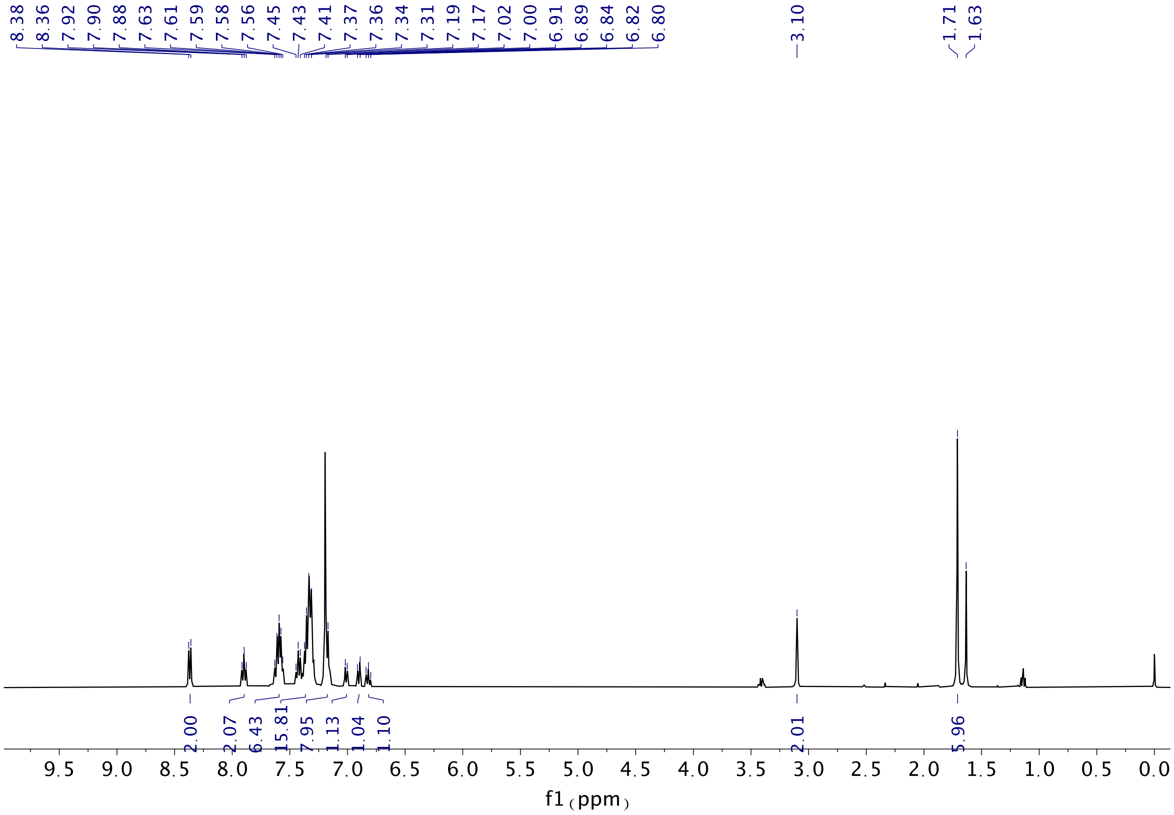

Figure S4: ^{1}H NMR spectrum of compound [**3**][Cl] in $CDCl_3$.

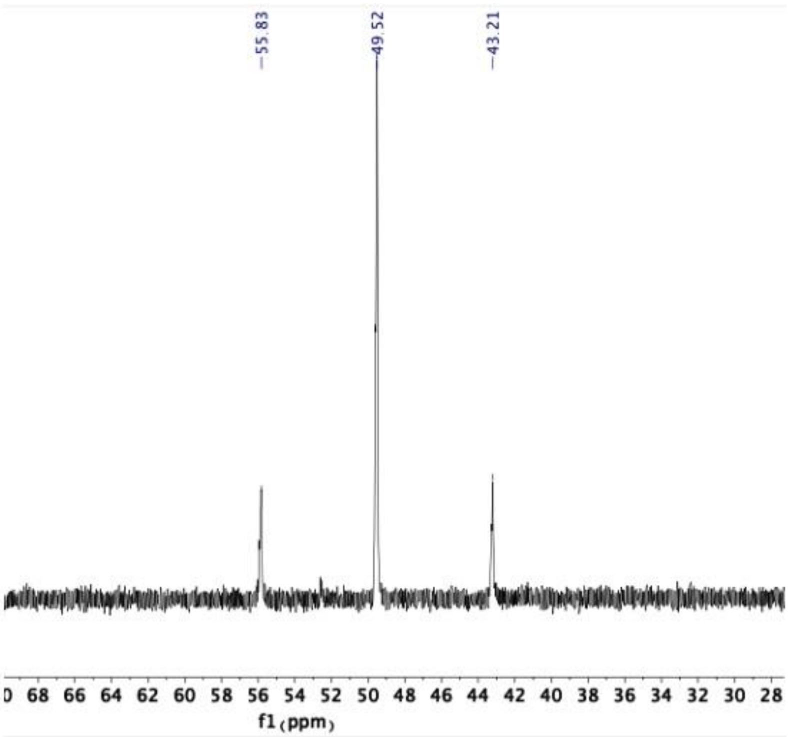

Figure S5: $^{31}P\{^1H\}$ NMR spectrum of compound [**3**][Cl] in $CDCl_3$.

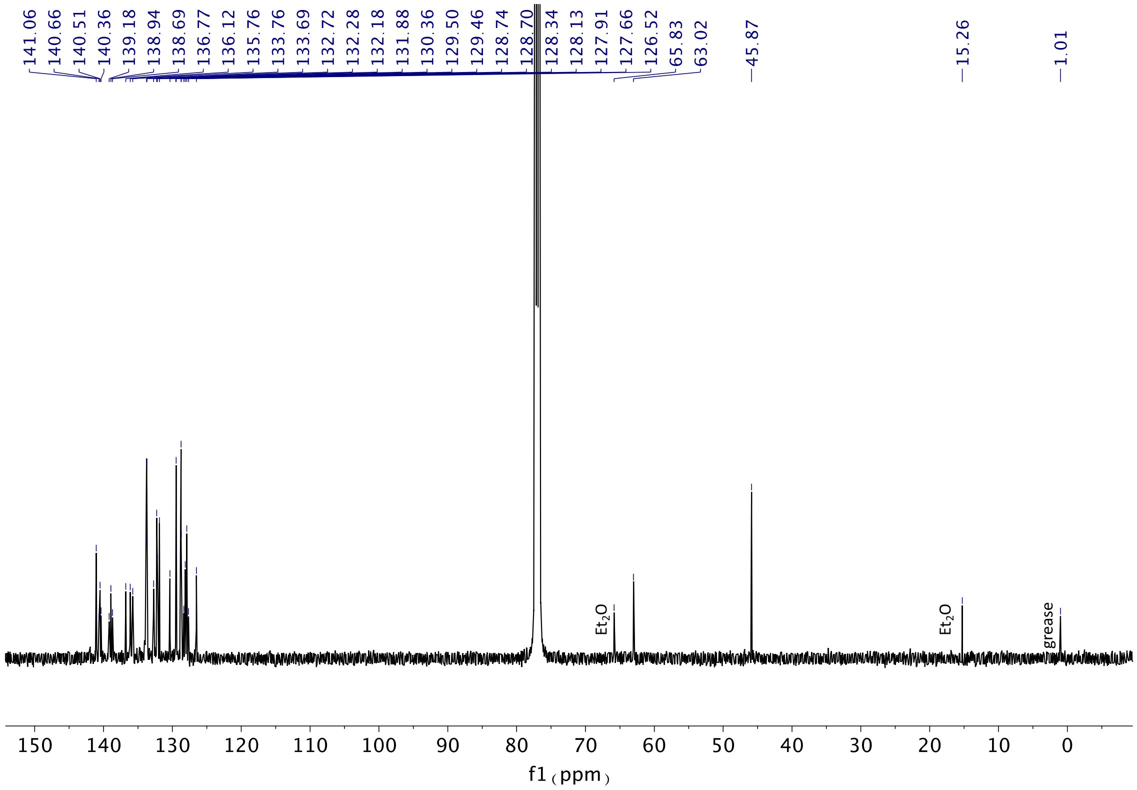

Figure S6: $^{13}C\{^1H\}$ NMR spectrum of compound [**3**][Cl] in $CDCl_3$.

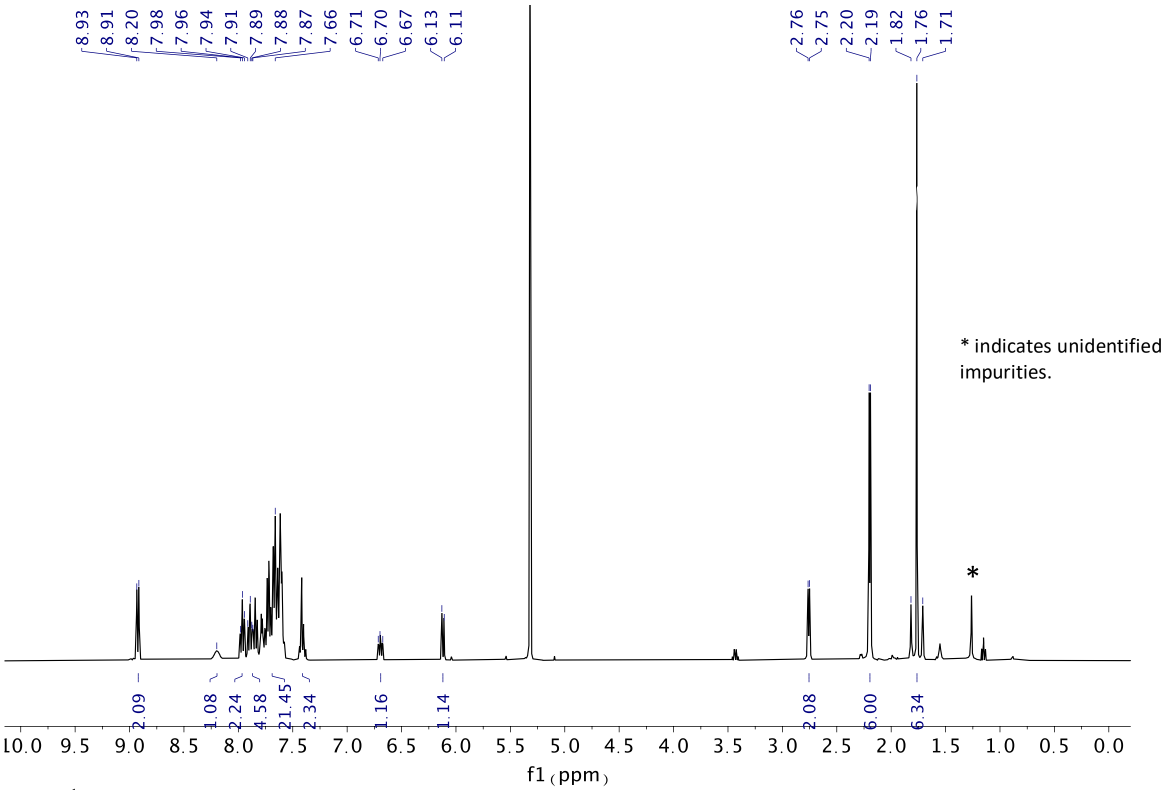

Figure S7:^{1}H NMR spectrum of compound [**4**][OTf]$_3$ in CD_2Cl_2.

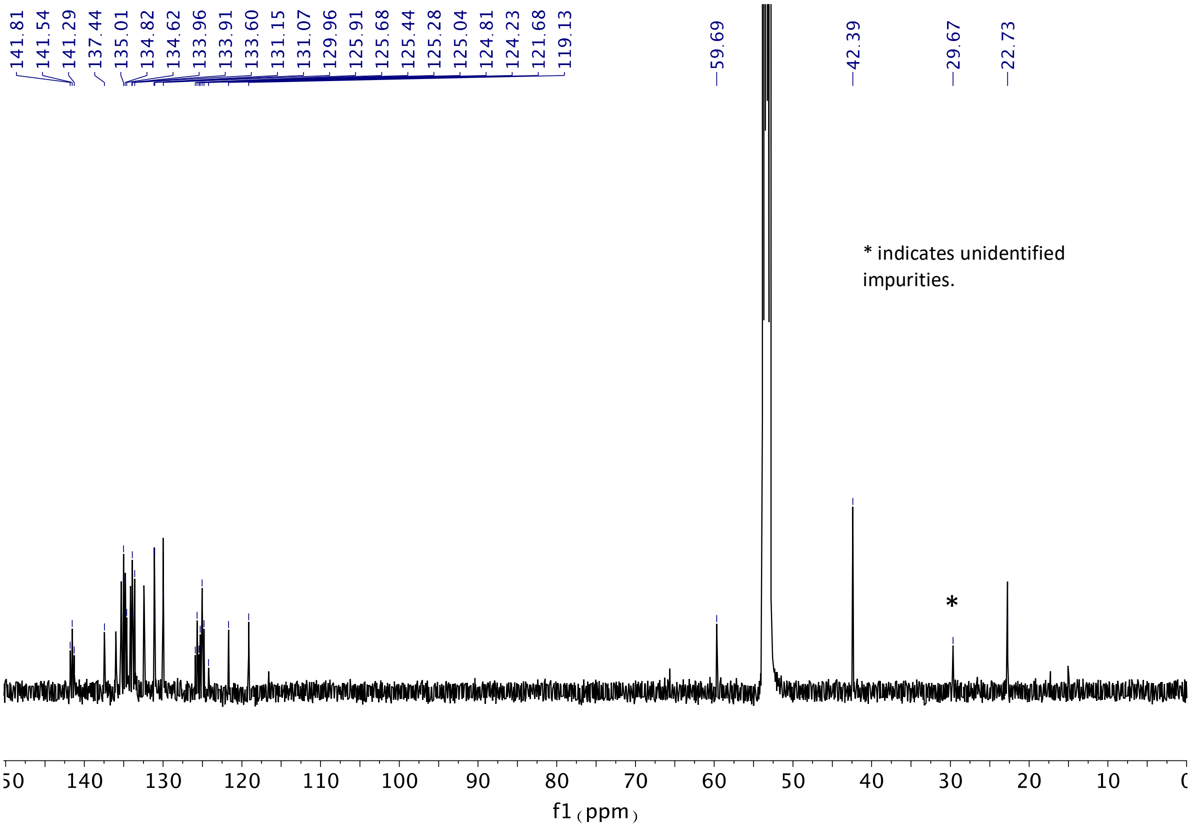

Figure S8: $^{13}C\{^1H\}$ NMR spectrum of compound [**4**][OTf]$_3$ in CD_2Cl_2.

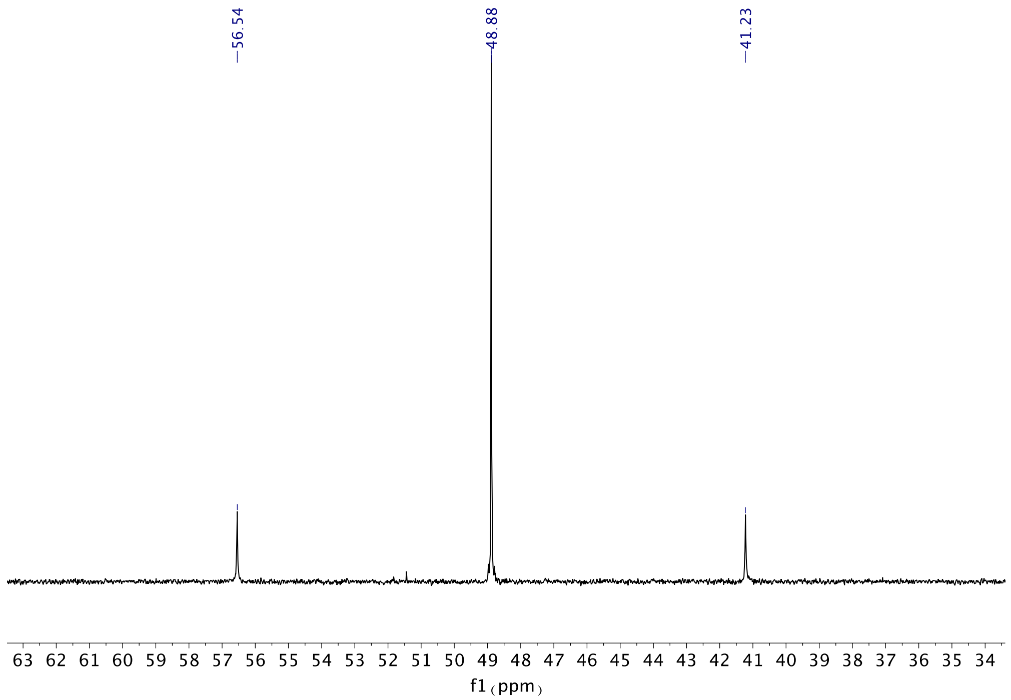

Figure S9: $^{31}P\{^1H\}$ NMR spectrum of [**4**][OTf]$_3$ in CD_2Cl_2.

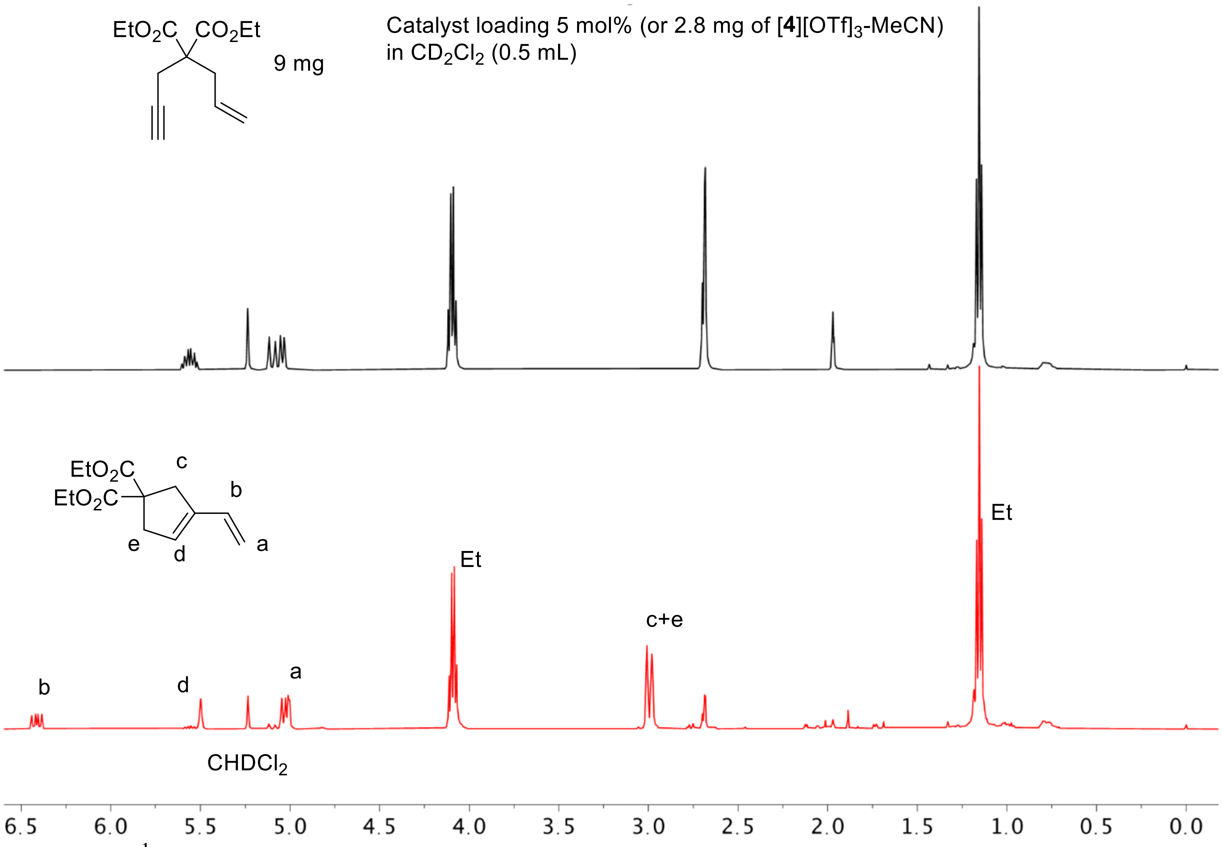

Figure S10: ^1H NMR spectra of collected during the cyclization of 2-allyl-2-(2-propynyl)malonate catalyzed by [**4**][OTf]$_3$ (5 mol%) in CD_2Cl_2 at 50°C.

Figure S11: Selected Natural Bond Orbitals involved in the N→Sb interaction present in the [**3**]$^+$.

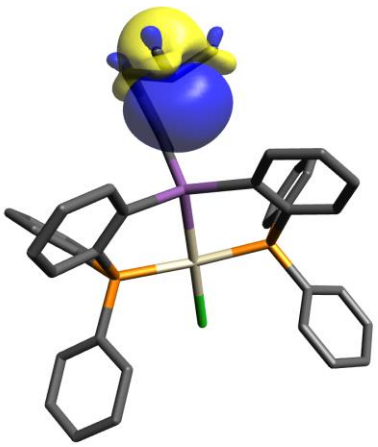

lp(N) (donor orbital)

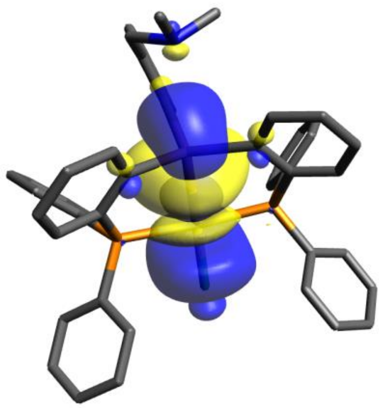

σ*(Sb-Pt) (acceptor orbital)

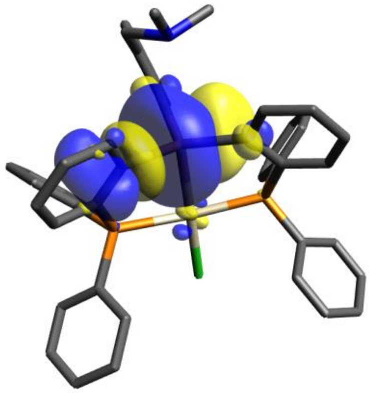

σ*(Sb-C)$_1$ (acceptor orbital)

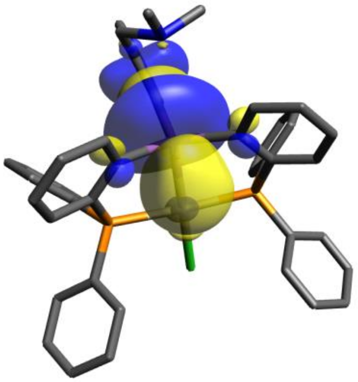

σ*(Sb-C)$_2$ (acceptor orbital)

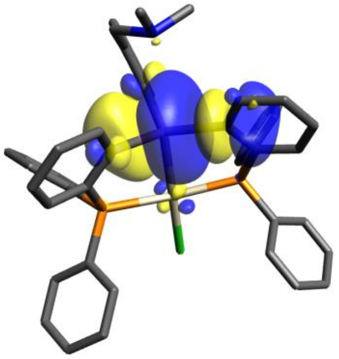

σ*(Sb-C)$_3$ (acceptor orbital)

Table S1. Cartesian coordinates (in Å) for the optimized structure of [**3**]$^+$.

Pt	0.0781853314	1.0349795375	0.2788834501
Sb	-0.0787199581	-1.2907165574	-0.6253187479
Cl	0.2099648345	3.2410240405	1.1944921592
P	-2.2279104598	1.0516318763	0.0015221493
P	2.3632930348	0.7602501464	0.0485562227
C	2.8921769361	0.7456136711	2.7586172549
H	2.1795082610	1.5626642702	2.7871456691
C	1.7600103741	-1.3962914957	-1.6916868443
C	2.7140013402	-0.4464257862	-1.2993435800
C	-2.9387105995	2.6597328628	-0.4770476758
C	-1.8460897997	-1.1082499006	-1.7913848199
C	-2.3332157771	3.3537585083	-1.5283430907
H	-1.4571152563	2.9430156784	-2.0174905250
C	3.2830973593	2.2517396270	-0.4508853239
C	-3.8717633993	0.1621972346	-2.1028430409
H	-4.5285707153	0.9760388804	-1.8200178154
N	-0.1285571981	-4.4411725167	-1.4715060241
C	-0.3248854165	-2.6706565673	0.9773349571
C	3.1980598625	0.1290016308	1.5409463031
C	2.7680054089	3.0286500288	-1.4922520303
H	1.8312403834	2.7530125221	-1.9632101623
C	3.9385692172	-0.4021985834	-1.9691626121
H	4.6846261853	0.3306195712	-1.6862343399
C	4.4789708932	2.6197750516	0.1639088186
H	4.8826088894	2.0272685344	0.9756338958
C	-0.0108764298	-2.1379612249	2.2270911030
H	0.3514267735	-1.1193883505	2.3082787774
C	3.2410294116	-2.2024779757	-3.4078284278
H	3.4359874870	-2.8723661130	-4.2361593600
C	-3.4592262776	0.5678501296	3.8474126486
H	-3.1465900499	0.9383308873	4.8160006610
C	-4.0540474582	3.1997321630	0.1606650866
H	-4.5271299457	2.6704456161	0.9785908534
C	-2.1737972907	-1.8983143671	-2.8857000240
H	-1.5051457028	-2.6824622092	-3.2100331848
C	-2.6886477722	-0.0591475902	-1.3957887244
C	2.0217476286	-2.2655531842	-2.7428852398
H	1.2733072728	-2.9825549263	-3.0481923632
C	3.4979769880	0.3088355765	3.9281677259
H	3.2613191453	0.7919615140	4.8682372513
C	-4.2547848948	-0.3886538704	1.3548334777
H	-4.5676889921	-0.7694737316	0.3906895141

C	5.1569953418	3.7559459080	-0.2652129031
H	6.0851343449	4.0389987380	0.2160441488
C	-2.7698644772	0.9559747614	2.7070734792
H	-1.9180920425	1.6224486845	2.7861480748
C	3.4526674807	4.1564894135	-1.9195751569
H	3.0506647109	4.7550103312	-2.7278839497
C	4.1999003864	-1.2783384992	-3.0147126812
H	5.1510758280	-1.2277720556	-3.5297570268
C	-3.1711943506	0.4843875182	1.4533988025
C	-4.2029818488	-0.6398145733	-3.1878766038
H	-5.1209949560	-0.4529583687	-3.7308030584
C	4.6472998877	4.5219962494	-1.3054153487
H	5.1775694607	5.4063900142	-1.6373671107
C	-3.3518867238	-1.6617166814	-3.5858841704
H	-3.5967766800	-2.2722099795	-4.4462028276
C	4.3983075223	-0.7516447725	3.8931822490
H	4.8649383765	-1.0957700128	4.8082391024
C	4.6939576538	-1.3736416687	2.6866470008
H	5.3899562572	-2.2030384325	2.6577247199
C	-2.8498129667	4.5712408560	-1.9442617605
H	-2.3788972959	5.1043220850	-2.7611224803
C	-0.1692909640	-2.9001518800	3.3787614064
H	0.0814724384	-2.4772684126	4.3437024512
C	-4.5627751637	4.4256201202	-0.2568791775
H	-5.4294232460	4.8427258298	0.2412870584
C	4.0986778301	-0.9353243247	1.5090704091
H	4.3340876929	-1.4277196317	0.5739212897
C	-4.9343063593	-0.7804767440	2.5024605551
H	-5.7724723727	-1.4617305283	2.4217896963
C	1.1318500293	-5.0312308960	-1.0238600162
H	1.5113572960	-4.5088165936	-0.1460174430
H	1.8748074054	-4.9517439390	-1.8171868391
H	1.0105753081	-6.0955599826	-0.7712252976
C	-0.6510693417	-4.1965118583	3.2765771922
H	-0.7812007536	-4.8025182311	4.1649432310
C	-0.8064642133	-3.9791965910	0.8623289644
C	-4.5400884319	-0.3021071465	3.7460917024
H	-5.0719953320	-0.6104822542	4.6379964867
C	-0.9607175227	-4.7253624373	2.0294209521
H	-1.3257887611	-5.7434270528	1.9537631002
C	-3.9649940621	5.1090763348	-1.3076796291
H	-4.3649337658	6.0626618822	-1.6306334948
C	-1.1675413704	-4.6049715902	-0.4567366512
H	-1.3851786950	-5.6709689873	-0.2883779328
H	-2.0877262632	-4.1529855354	-0.8423302993
C	-0.5551510619	-5.1132194219	-2.6967896504
H	0.1599397979	-4.9262347453	-3.4996356675
H	-1.5356910284	-4.7536723067	-3.0088767933
H	-0.6288753850	-6.2019518012	-2.5537605494
